# Gender specific factors contributing to cognitive resilience in *APOE ɛ4* positive older adults in a population-based sample

**DOI:** 10.1038/s41598-023-34485-1

**Published:** 2023-05-17

**Authors:** Lidan Zheng, Ranmalee Eramudugolla, Nicolas Cherbuin, Shannon M. Drouin, Roger A. Dixon, Kaarin J. Anstey

**Affiliations:** 1 School of Psychology, The University of New South Wales, Sydney, NSW Australia; 2 Neuroscience Research Australia (NeuRA), Randwick, NSW Australia; 3 UNSW Ageing Futures Institute, Kensington, NSW Australia; 4 Centre for Research on Ageing, Health and Wellbeing, Australian National University, Canberra, ACT Australia; 5 Department of Psychology, University of Alberta, Edmonton, Canada; 6 Neuroscience and Mental Health Institute, University of Alberta, Edmonton, Canada

**Keywords:** Risk factors, Predictive markers, Human behaviour

## Abstract

Although *APOE ɛ4* has been identified as the strongest genetic risk factor for Alzheimer’s Disease, there are some *APOE ɛ4* carriers who do not go on to develop Alzheimer’s disease or cognitive impairment. This study aims to investigate factors contributing to this “resilience” separately by gender. Data were drawn from *APOE ɛ4* positive participants who were aged 60 + at baseline in the Personality and Total Health Through Life (PATH) Study (N = 341, Women = 46.3%). Participants were categorised into “resilient” and “non-resilient” groups using Latent Class Analysis based on their cognitive impairment status and cognitive trajectory across 12 years. Logistic regression was used to identify the risk and protective factors that contributed to resilience stratified by gender. For *APOE ɛ4* carriers who have not had a stroke, predictors of resilience were increased frequency of mild physical activity and being employed at baseline for men, and increased number of mental activities engaged in at baseline for women. The results provide insights into a novel way of classifying resilience among *APOE ɛ4* carriers and risk and protective factors contributing to resilience separately for men and women.

## Introduction

Apolipoprotein (*APOE) ɛ4* is the strongest identified genetic risk factor for Alzheimer’s Disease (AD)^1^. It has been associated with cholesterol metabolism and the clearing of beta-amyloid from the brain^1^. Carrying one *APOE ɛ4* allele (heterozygous) has been shown to increase the risk of developing AD by approximately 2–3 fold compared to carriers of two *APOE ɛ3* alleles, while carrying two *APOE ɛ4* alleles (homozygous) has been shown to increase the risk by almost 15 fold^2,3^.

Despite this increased risk, there are some *APOE ɛ4* carriers who remain cognitively normal and do not go on to develop dementia or cognitive impairment even in very old age^4^. This “cognitive resilience” may be attributed to both genetic (e.g. interactions with protective genes or risk-reducing alleles such as *APOE ɛ2*^1^) as well as numerous modifiable lifestyle and medical factors that can decrease the risk of *APOE ɛ4* carriers developing dementia or clinically significant cognitive impairment^5,6^. Recent research has shown that modifiable risk factors could prevent or delay up to 40% of dementias^7^. Factors that decrease risk (also known as protective factors) include more years of education, Mediterranean-like diets, physical activity, social activity and cognitive activity^7,8^. Notably, factors that can increase risk include smoking, high alcohol consumption, and the presence of conditions such as diabetes, hypertension, stroke, heart problems and high cholesterol^7,8^.

While several different definitions have been proposed^9,10^, in general “cognitive resilience” in the context of cognitive aging refers to maintained cognitive performance despite adversities normally associated with exacerbated cognitive decline or impairment^11^. These can include preclinical dementia neuropathology^9^, as well as specific dementia risk factors such as brain injury and genetic risk^12^.

So far (to this author’s knowledge) there have only been four studies^12–15^ that have looked at factors contributing to cognitive resilience in *APOE ɛ4* positive individuals. These four studies have used two main operationalisations of “cognitive resilience”: (1) The absence of dementia or mild cognitive impairment (MCI) in older age^13,14^ and (2) cognitive performance or trajectory in *APOE ɛ4* carriers (e.g. cognitive performance in the highest tertile among *APOE ɛ4* carriers)^12,15^. Using the first operationalisation, Ferrari et al.^13^ examined factors that reduced dementia risk in *APOE ɛ4* carriers aged over 75 across 9 years (n = 932). They found that higher education, high level of leisure activities and absence of vascular risk factors contributed to reduced dementia risk after adjusting for demographic and health covariates. Similarly, Hayden et al.^14^ examined risk and protective factors among women (N = 557, age 65–79 at enrolment) who were *APOE ɛ4* carriers. They found that higher self-rated general health and cognitive scores predicted surviving to age 80+ without cognitive impairment after controlling for a range of demographic, health and lifestyle factors.

On other hand, using the second operationalisation, Kaup et al. examined differences between factors that contributed to cognitive resilience in black (N = 329) and white (N = 341) older adults who were *APOE ɛ4* carriers^12^. They defined cognitive resilience as a cognitive trajectory that fell within the highest tertile compared with demographically similar individuals. They used random forest analysis to examine a pool of 23 potential predictors of resilience, the results identified five important predictors of cognitive resilience among white individuals (i.e. absence of recent negative life events, literacy level, older age, educational level, and time spent reading) and four important predictors among black individuals (i.e. literacy level, educational level, sex and absence of diabetes). Using a similar operational approach, McDermott et al. examined sex differences in memory resilience prediction profiles in people with genetic risk (*APOE* and *Clusterin*)^15^. They defined cognitive resilience based on objective evidence of maintained episodic memory performance trajectories in the context of genetic risk using growth mixture models. Following this up with random forest analysis, they found 14 important predictors of cognitive resilience out of the 22 predictors they included in their analysis, including less depressive symptoms for males; living with someone, being married, lower pulse pressure, higher peak expiratory flow, faster walking time, faster turning time, more social visits and volunteering more often for females; and younger age, higher education, stronger grip and more cognitive activity for both sexes.

Overall, we believe there are merits to both types of operationalisations of “cognitive resilience”. As this is a relatively new area of research, we aim to contribute to the growing evidence base and expand the current definitions by exploring whether a combined definition of cognitive resilience (based on both cognitive trajectory and dementia status), may lead to new insights into factors that contribute to cognitive resilience in *APOE ɛ4* carriers. In addition, rather than using a priori criteria to define “resilience” we will used an objective data-driven (unsupervised clustering) technique, specifically latent class analysis (LCA), to classify participants. LCA is generally useful for when there is high variability in the data and the number of groups is unknown^16^.

An added layer of complexity in this area of research is the consideration of gender differences in the aetiology of dementia and dementia risk factors. Recently published research has shown that modifiable risk factors may differ between men and women^17^ and complex *APOE* genotype by sex and gender interaction effects can occur across various pathological, physiological and lifestyle risk factors such as physical activity, pulse pressure and AD pathology^18–20^. As such, and in line with recent recommendations in the field^21^, this paper will stratify the results by gender when exploring modifiable risk factors for cognitive resilience among *APOE ɛ4* carriers.

In sum, this study aims to (1) identify cognitively resilient groups of people who are *APOE ɛ4* positive using both cognitive trajectory and cognitive impairment (MCI and dementia) status, and (2) identify clinical and lifestyle factors which contribute to resilience stratified by gender.

Specifically, we hypothesise that:

- Hypothesis 1: Resilient and non-resilient groups will emerge among people who are *APOE ɛ4* positive when using both cognitive trajectory and cognitive impairment status as part of the classification criteria.
- Hypothesis 2: The resilient group/groups will score higher in protective factors and lower in risk factors for cognitive decline and dementia compared to the non-resilient groups.

## Results

### Latent class analysis

The results showed that two classes significantly improved fit over one class (LMR p = < 0.001) while a third class only marginally improved fit over two classes (LMR p = 0.045) (see Appendix A). When plotting the relative fit indices (AIC, BIC, SABIC), an elbow can be observed at two classes. Therefore, using the parsimony principle^22^, we chose to proceed using the two class model (Class 1: Full Sample N = 586, *APOE ɛ4* carriers N = 101; Class 2: Full Sample N = 1515, *APOE ɛ4* carriers N = 240). Post-hoc examination of the groups using *t* tests showed no difference in immediate recall performance at wave 1 (Group 1 mean = 7.65 (SD = 2.14); Group 2 mean = 7.17 (SD = 2.67); p = 0.106) but indicated that group 1 performed significantly better in immediate recall at wave 4 (Group 1 mean = 7.57 (SD = 1.32); Group 2 mean = 4.36 (SD = 1.31); p = < 0.001). Examination of the incidence of cognitive impairment across the two groups showed that the prevalence of cognitive impairment at wave 4 in group 1 (henceforth known as the “resilient” group) was 1.64%, compared to 36.54% in group 2 (henceforth known as the “non-resilient” group). When examining cognitive impairment prevalence specifically in individuals who were *APOE ɛ4* carriers, there was only one participant with cognitive impairment at wave 4 in group 1. Further examination showed that this individual had a stroke at wave 4. To control for cognitive impairment caused by stroke, we excluded all participants who had suffered a stroke at any point (wave 4 or prior) in the subsequent analysis (N = 33). To assess for any biases as a result of excluding these participants, we conducted a sensitivity analysis with individuals with stroke.

Descriptive statistics of both groups are presented in Table 1 for individuals who are *APOE ɛ4* carriers. The descriptive statistics and demographics of *non-APOE ɛ4* carriers in the wider 60s PATH cohort are also included in Appendix B.

**Table 1:** Descriptive statistics of the resilient and non-resilient groups (after excluding stroke).

	Group 1 (Cognitive Resilient Group, N = 91)	Group 2 (Non-Resilient Group, N = 212)	T-value (Cohen’s d)/Chi-squared (Cramer’s V)	p-value
Mean age (wave 1)	62.32 (1.41)	62.33 (1.47)	0.063 (0.008)	0.950
Immediate recall (wave 4)	**7.59 (1.34)**	**4.43 (1.29)**	**16.889 (2.418)**	**< 0.001**
Dementia/MCI diagnoses (wave 4)	**0**	**49 (29%)**	**25.867 (0.328)**	**< 0.001**
Years of education (wave 1)	**14.98 (2.32)**	**14.07 (2.48)**	− **2.976 (0.373)**	**0.003**
Women	49 (53.8%)	93 (43.9%)	2.546 (0.092)	0.111
Partnered (wave 1)	81 (89.0%)	177 (83.5%)	1.534 (0.071)	0.215
Employed (wave 1)	44 (48.4%)	87 (41.0%)	1.388 (0.068)	0.239
APOE allele status				
ɛ2/ɛ4	11 (12.1%)	22 (10.4%)	0.296 (0.031)	0.863
ɛ3/ɛ4	75 (82.4%)	180 (84.9%)		
ɛ4/ɛ4	5 (5.5%)	10 (4.7%)		
Hypertension (wave 1)	55 (60.4%)	125 (59.0%)	0.058 (0.014)	0.810
Cholesterol medication (wave 1)	22 (24.2%)	49 (23.1%)	0.040 (0.011)	0.841
Diabetes (wave 1)	6 (6.6%)	22 (10.4%)	1.087 (0.060)	0.297
Heart problems (wave 1)	9 (9.9%)	21 (9.9%)	0.000 (0.000)	0.997
Head injury (wave 1)	1 (1.1%)	11 (5.2%)	2.800 (0.096)	0.094
BMI (wave 1)	26.28 (5.08)	26.39 (4.31)	0.201 (0.025)	0.841
MIND diet (wave 1)	6.76 (1.44)	6.42 (1.38)	− 1.975 (− 0.248)	0.053
Frequency of physical activity (wave 1)				
Mild	3.76 (0.6)	3.61 (0.73)	− 1.799 (− 0.209)	0.074
Moderate	2.93 (1.01)	2.94 (1)	0.037 (0.005)	0.971
Vigorous	1.62 (0.92)	1.75 (0.99)	1.151 (0.144)	0.251
Anxiety score (wave 1)	1.96 (2.2)	2.01 (2.17)	0.213 (0.027)	0.832
Depression score (wave 1)	1.24 (1.5)	1.44 (1.69)	1.032 (0.123)	0.303
Number of cigarettes per day (wave 1)	1.96 (2.2)	2.01 (2.17)	− 0.219 (− 0.027)	0.827
Number of drinks per week (wave 1)	1.24 (1.5)	1.44 (1.69)	− 0.932 (− 0.117)	0.352
Net positive social interactions (wave 1)	12.16 (8.01)	12.07 (7.75)	− 0.101 (− 0.013)	0.920
Mental activity (wave 1)	23.99 (6.34)	22.36 (7.55)	− 1.929 (− 0.226)	0.055

Significant values are in bold.

Chi-squared and independent sample *t* tests were used to compare protective and risk factors between the resilient and non-resilient groups. Years of education was found to be significantly higher in the resilient group (p = 0.003). MIND diet score (p = 0.053, Cohen’s d = − 0.248) and number of mental activities (p = 0.055, Cohen’s d = − 0.248) were also higher in the resilient group but had small effect sizes and were not statistically significant.

### Predictors of resilience stratified by gender

*T* tests were used to determine differences between baseline characteristics of the two resilience groups stratified by gender (Tables 2 and 3). Years of education, and levels of vigorous physical activity were significantly different between the resilient group and non-resilient group for men, while years of education and mental activity were significantly different for women.

**Table 2:** Bivariate comparisons and logistic regression results for men (N = 161).

*T* test/Chi-squared					Odds ratio^†^ (95% CI)		
Variables	Resilient (N = 42)	Non-resilient (N = 119)	T-value (Cohen’s d)/Chi-squared (Cramer’s V)	p-value	Model 1 (Protective Factors only)	Model 2 (Including demographic and genetic risk factors)	Model 3 (Including medical and lifestyle risk factors)
Years of education (wave 1)	**15.43 (2.4)**	**14.32 (2.57)**	− **2.446 (**− **0.439)**	**0.016**	1.194 (0.998; 1.429)	1.186 (0.987; 1.425)	1.197 (0.969; 1.48)
MIND diet score	6.62 (1.5)	6.22 (1.45)	− 1.506 (− 0.270)	0.134	1.061 (0.789; 1.426)	1.144 (0.834; 1.569)	1.274 (0.876; 1.852)
Frequency of physical activity (wave 1)							
Mild	3.74 (0.66)	3.56 (0.76)	− 1.415 (− 0.239)	0.161	1.633 (0.852; 3.13)	1.908 (0.968; 3.763)	2.439 (1.161; 5.124)*
Moderate	3.17 (0.79)	3.11 (0.88)	− 0.372 (− 0.067)	0.710	1.045 (0.625; 1.746)	0.933 (0.543; 1.603)	0.818 (0.441; 1.516)
Vigorous	**1.52 (0.8)**	**1.93 (1.03)**	**2.331 (0.418)**	**0.021**	0.532 (0.33; 0.859)**	0.512 (0.316; 0.831)**	0.45 (0.259; 0.784)**
Positive social interactions (wave 1)	12.62 (6.62)	13.38 (7.38)	0.588 (0.106)	0.557	0.999 (0.946; 1.054)	1.008 (0.951; 1.069)	1.012 (0.937; 1.092)
Mental activity (wave 1)	25.67 (6.79)	24.44 (7.36)	− 0.949 (− 0.170)	0.344	1.008 (0.95; 1.07)	0.995 (0.933; 1.062)	1.012 (0.94; 1.089)
Age (wave 1)	62.48 (1.49)	62.36 (1.52)	− 0.424 (− 0.076)	0.672	–	1.108 (0.856; 1.434)	1.114 (0.835; 1.485)
Partnered (wave 1)	40 (95.2%)	112 (94.1%)	0.074 (0.021)	0.786	–	0.733 (0.104; 5.171)	0.636 (0.078; 5.161)
Employed (wave 1)	24 (57.1%)	52 (43.7%)	2.252 (0.118)	0.133	–	2.108 (0.932; 4.767)	2.479 (1.007; 6.102)*
*APOE* allele status							
* APOE ɛ2/ɛ4*	4 (9.5%)	12 (10.1%)	2.240 (0.118)		–	0.642 (0.176; 2.343)	0.794 (0.188; 3.346)
* APOE ɛ4/ɛ4*	0	6 (5.0%)		0.326	–	0	0
Hypertension (wave 1)	30 (71.4%)	76 (63.9%)	0.789 (0.070)	0.374	–	–	1.956 (0.72; 5.31)
Cholesterol medication (wave 1)	14 (33.3%)	28 (23.5%)	1.547 (0.098)	0.214	–	–	1.594 (0.555; 4.583)
Diabetes (wave 1)	1 (2.4%)	12 (10.1%)	2.482 (0.124)	0.115	–	–	0.216 (0.023; 2.039)
Heart problems (wave 1)	6 (14.3%)	14 (11.8%)	0.181 (0.034)	0.670	–	–	1.313 (0.323; 5.334)
Head injury (wave 1)	1 (2.4%)	9 (7.6%)	1.431 (0.094)	0.232	–	–	0.212 (0.019; 2.367)
BMI (wave 1)	26.43 (3.02)	26.66 (3.95)	0.350 (0.063)	0.727	–	–	0.900 (0.793; 1.022)
Anxiety score (wave 1)	2.07 (2.3)	1.71 (1.98)	− 0.984 (− 0.177)	0.327	–	–	1.124 (0.832; 1.52)
Depression score (wave 1)	1.36 (1.67)	1.3 (1.6)	− 0.188 (− 0.034)	0.851	–	–	1.027 (0.715; 1.474)
Number of cigarettes per day (wave 1)	1.74 (5.97)	0.84 (4.43)	− 1.026 (− 0.184)	0.306	–	–	1.092 (0.995; 1.198)
Number of drinks per week (wave 1)	8.39 (6.74)	7.8 (7.5)	− 0.449 (− 0.081)	0.654	–	–	0.979 (0.917; 1.046)

*p < 0.05; **p < 0.01. ^†^Non-resilient group used as reference group. Model 2 includes *APOE ɛ4* allele status, age, partnered and employment status. Model 3 includes hypertension, diabetes, heart problems, head injury, BMI, cholesterol medication, anxiety, depression, smoking and alcohol consumption.

Significant values are in bold.

**Table 3:** Bivariate comparisons and logistic regression results for women (N = 142).

*T* test/Chi-squared					Odds ratio^†^ (95% CI)		
Variables	Resilient (N = 49)	Non-resilient (N = 93)	T-value (Cohen’s d)/Chi-squared (Cramer’s V)	p-value	Model 1 (Protective Factors only)	Model 2 (Including demographic and genetic risk factors)	Model 3 (Including medical and lifestyle risk factors)
Years of education (wave 1)	**14.59 (2.19)**	**13.75 (2.34)**	− **2.077 (**− **0.367)**	**0.040**	1.113 (0.94; 1.317)	1.082 (0.905; 1.293)	1.066 (0.883; 1.286)
MIND diet score	6.89 (1.38)	6.67 (1.25)	− 0.966 (− 0.171)	0.336	1.120 (0.832; 1.508)	1.133 (0.831; 1.544)	1.097 (0.788; 1.527)
Frequency of physical activity (wave 1)							
Mild	3.78 (0.55)	3.68 (0.69)	− 0.857 (− 0.151)	0.393	1.157 (0.603; 2.222)	1.165 (0.583; 2.328)	0.967 (0.463; 2.022)
Moderate	2.73 (1.13)	2.72 (1.11)	− 0.072 (− 0.013)	0.942	0.852 (0.586; 1.239)	0.876 (0.6; 1.28)	0.873 (0.582; 1.311)
Vigorous	1.69 (1)	1.53 (0.88)	− 1.024 (− 0.181)	0.308	1.328 (0.865; 2.038)	1.304 (0.841; 2.023)	1.307 (0.81; 2.111)
Positive social interactions (wave 1)	11.78 (9.09)	10.39 (7.94)	− 0.942 (− 0.166)	0.348	1.017 (0.971; 1.065)	0.996 (0.946; 1.049)	0.996 (0.941; 1.055)
Mental activity (wave 1)	**22.55 (5.61)**	**19.71 (6.96)**	− **2.465 (**− **0.435)**	**0.015**	1.063 (1.002; 1.127)*	1.064 (1.002; 1.131)*	1.069 (1.001; 1.141)*
Age (wave 1)	62.18 (1.35)	62.29 (1.42)	0.433 (0.076)	0.666	–	0.914 (0.695; 1.202)	0.918 (0.687; 1.225)
Partnered (wave 1)	41 (83.7%)	65 (69.9%)	3.220 (0.151)	0.073	–	2.185 (0.773; 6.176)	2.187 (0.705; 6.781)
Employed (wave 1)	20 (40.8%)	35 (37.6%)	0.137 (0.031)	0.711	–	0.902 (0.398; 2.044)	0.781 (0.332; 1.839)
*APOE* allele status							
* APOE ɛ2/ɛ4*	7 (14.3%)	10 (10.8%)	2.449 (0.131)		–	1.384 (0.461; 4.15)	1.437 (0.463; 4.466)
* APOE ɛ4/ɛ4*	5 (10.2%)	4 (4.3%)		0.294	–	2.322 (0.511; 10.559)	1.814 (0.382; 8.613)
Hypertension (wave 1)	25 (51.0%)	49 (52.7%)	0.036 (0.016)	0.850	–	–	1.087 (0.489; 2.417)
Cholesterol medication (wave 1)	8 (16.3%)	21 (22.6%)	0.772 (0.074)	0.379	–	–	0.670 (0.23; 1.952)
Diabetes (wave 1)	5 (10.2%)	10 (10.8%)	0.010 (0.008)	0.919	–	–	0.694 (0.171; 2.818)
Heart problems (wave 1)	3 (6.1%)	7 (7.5%)	0.097 (0.026)	0.756	–	–	0.543 (0.107; 2.739)
Head injury (wave 1)	0	2 (2.2%)	1.069 (0.087)	0.301	–	–	0
BMI (wave 1)	26.15 (6.37)	26.04 (4.73)	− 0.110 (− 0.019)	0.912	–	–	1.034 (0.955; 1.119)
Anxiety score (wave 1)	1.86 (2.13)	2.41 (2.34)	1.377 (0.243)	0.171	–	–	0.942 (0.735; 1.207)
Depression score (wave 1)	1.14 (1.35)	1.62 (1.79)	1.795 (0.291)	0.075	–	–	0.963 (0.66; 1.406)
Number of cigarettes per day (wave 1)	0.51 (2.93)	1.11 (3.98)	0.925 (0.163)	0.357	–	–	0.995 (0.867; 1.143)
Number of drinks per week (wave 1)	5.66 (5.53)	4.04 (4.86)	− 1.800 (− 0.318)	0.074	–	–	1.049 (0.969; 1.135)

*p < 0.05. ^†^Non-resilient group used as reference group. Model 2 includes *APOE ɛ4* allele status, age, partnered and employment status. Model 3 includes hypertension, diabetes, heart problems, head injury, BMI, cholesterol medication, anxiety, depression, smoking and alcohol consumption.

Significant values are in bold.

The proportion of men with diabetes (Resilient = 2.4%, Non-resilient = 10.1%, Cramer’s V = 2.482) and head injury (Resilient = 2.4%, Non-resilient = 7.6%, Cramer’s V = 1.431) at baseline were also slightly higher in the non-resilient group compared to the resilient group, however did not reach statistical significance with small to trivial effect sizes. Similarly, the proportion of women with head injury (Resilient = 0%, Non-resilient = 2.2%, Cramer’s V = 1.069), partnered status (Resilient = 83.7%, Non-resilient = 69.9%, Cramer’s V = 3.220), mean depression score (Resilient = 1.14, Non-resilient = 1.62, Cohen’s d = 1.795) and mean number of drinks per week (Resilient = 5.66, Non-resilient = 4.04, Cohen’s d = − 1.800) appeared different between the two groups but did not reach significance and had small to trivial effect sizes.

Logistic regression analyses showed that less frequent vigorous physical activity, more frequent mild physical activity and being employed at baseline (around age 60) were independent predictors of cognitive resilience over 12 years for men whereas increased mental activity predicted cognitive resilience for women. Specifically, men who reported more frequent mild physical activity and being employed at baseline were 2.4 and 2.5 times more likely to be in the resilient group respectively, while women who reported participating in more mental activity were 1.1 times more likely to be in the resilient group. On the opposite end men who reported more frequent vigorous physical activity were 2.2 times more likely to be in the “non-resilient” group.

*APOE* allele status (i.e. homozygous, heterozygous and the presence of the *ɛ2* allele) was not found to be a significant predictor in either men or women.

### Sensitivity analysis

Sensitivity analysis was conducted by including people who have ever had a stroke in the analysis (see Appendix C, men N = 22, women N = 16). The results of gender comparisons at baseline between the two groups remained the same in men and women, with the addition of depression showing a baseline difference in women (more depressive symptoms in the non-resilient group). The results of the binary logistic regression showed that years of education became significant while employment at baseline was no longer significant for men and there were no significant independent predictors for women.

## Discussion

To investigate hypothesis 1, we used memory performance and dementia status to identify a ‘cognitively resilient’ group and ‘non-resilient’ group in *APOE ɛ4* positive adults aged over 60. Our results showed that, in line with previous studies^12,13,15^, years of education was found to be significantly higher in the resilient group. While adherence to the MIND diet and number of mental activities were also found to be slightly higher in the resilient group, the effect sizes were small and not statistically significant. Future studies with larger sample sizes may help power these effects.

To investigate hypothesis 2, we examined protective factors and risk factors that contributed to resilience independently by gender. Our hypothesis was partially supported. We found that being employed at age 60 predicted resilience status for men while higher levels of mental activity predicted resilience status for women. It is possible that these two protective factors tap into the same mechanism of cognitive stimulation and that statistical differences in significant predictors was the result of differences in the employment rate between men and women in this sample (e.g. there were around 17% less women who were employed at age 60 in the resilient group compared to men).

More physical activity is a well-known protective factor against dementia^23^. However, an unexpected finding in this study was that more self-reported vigorous physical activity was inversely related to resilience status in men (while the opposite trend was found in women, this was not statistically significant and evaluation of the effect sizes showed that these effects were trivial). For men, it appears that more self-reported mild (rather than vigorous) physical activity predicted resilience status. The authors speculated that this could be due the effects of occupation where more vigorous physical activity is related to more laborious and physically demanding occupational roles; however, post-hoc analysis showed that there was no difference in frequency of vigorous physical activity between labour intensive roles and non-labour intensive roles in men (Chi-squared = 2.32, p = 0.509; see Appendix D). While this study included a number of lifestyle and demographic factors as covariates, it is possible that the underlying explanation for this effect may be biological, and thus beyond the scope of this study. Further research and replication may be necessary to understand why mild physical activity may be more beneficial for cognitive resilience than vigorous physical activity in older *APOE ɛ4* positive men. This finding may also be confounded by other variables associated with physically active time vs physically inactive time such as sedentary behaviour and/or sleep which have also been linked to cognition^24^. While we only have self-report data on physical activity in this dataset, recent advances in technology mean that it is possible to objectively measure daily activity and time use with through wearable devices which may help untangle these effects (e.g.^25^).

Sensitivity analysis was conducted by including people with stroke in the analysis. This resulted in changes in predictors for both men and women. Specifically for men, the inclusion of individuals with stroke helped power the effect of years of education in predicting resilience. However, the effect of employment was erased, indicating that being employed at baseline was not protective for men who have had a stroke within the last 12 years. In regards to women, when including individuals with stroke in the analysis, the non-resilient group were found to have more depressive symptoms. This is in accordance with research that shows that women are more likely to develop post-stroke depression^26,27^. Additionally, the inclusion of people with stroke erased the effect of mental activity as an independent predictor of cognitive resilience for women. This indicates that more mental activity at baseline is not protective for women after 12 years if they suffer a stroke.

### Operationalisation and mechanisms of cognitive resilience

Previous studies have employed two main operationalisations of cognitive resilience in *APOE ɛ4* positive adults (i.e. absence of dementia or cognitive performance/trajectory). Our study combines both operationalisation approaches and in support of hypothesis 1, was able to identify individuals who demonstrated cognitive resilience among *APOE ɛ4* carriers. We found some predictors that aligned with previous research on resilience in *APOE ɛ4* carriers (e.g. education^12,13,15^, mental activity in women^13,15^), and some that were different (e.g. staying employed and increased mild physical activity in men^12,14^). All in all, however, the results of this study are attuned with the existing evidence on cognitive resilience and dementia risk reduction^28^. The protective factors found in this study (e.g. physical and mental activity) are theorised to build cognitive reserve, which could counteract the effects of pathology in or damage to the brain, leading to resilience^29^. Additionally, there is some evidence showing that these factors can help older adults maintain their cognitive status and make them resistant to the accumulation of pathology in the brain^30,31^. Future neurobiological studies could evaluate the accuracy of our classification algorithm (i.e. using dementia status and cognitive trajectory) and examine whether the resilient group indeed has less neuropathology. As well as whether the inclusion of neurobiological markers (e.g. amyloid accumulation) within the classification algorithm affects the predictors found.

Interestingly, in contrast to our predictions in hypothesis 2, we did not find any dementia risk factors (e.g. medical and health conditions) to be related to resilience. This could be related in part to our statistical approach and sample size limitations. For example, LCA assigns individuals to classes based on their probability of being in classes so it can be subject to some classification errors. Additionally, our sample sizes became smaller once the sample was stratified into gender and resilient/non-resilient groups which may have resulted in some effects being underpowered (e.g. differences in adherence to the MIND diet and number of mental activities between resilience groups did not reach statistical significance). This is especially relevant considering that many of these risk/protective factors share variance. Alternatively, these results suggest that increasing protective factors may have a greater impact on improving resilience than lowering risk factors. Overall, more intervention and longitudinal studies are needed to fully understand the mechanisms by which these factors contribute to cognitive resilience. Future studies should also explore whether life-stage and duration of exposure related to these factors can influence one’s ability to develop or build cognitive resilience as well as whether targeting multiple overlapping factors will provide synergistic effects on cognition.

### Strengths and limitations

The strengths of this paper are that it (1) used a powerful data-driven approach (LCA) to detect subgroups from a heterogeneous population, (2) examined the utility of a combined conceptualisation of resilience based on both cognitive trajectory and dementia status and (3) examined whether other protective factors not included in previous studies such as diet, contributed to resilience in *APOE ɛ4* positive individuals. Limitations of our study include the use of a largely Caucasian sample drawn from a relatively well educated Australian urban population which may limit generalisability. Similarly, socioeconomic status (SES) may also influence some of these effects. Specifically, while we did not have data on income in this study, future studies could examine whether complex relationship between education, employment and SES could have an impact on the results of this study. Lastly, as with many cohort studies, our study relied on self-reported data for lifestyle risk factors which may be influenced by retrospective memory effects.

### Summary

Overall, our results showed that for *APOE ɛ4* carriers who have not had a stroke, staying employed and increased self-reported mild physical activity (rather than vigorous physical activity) predicted cognitive resilience for men, while increased mental activity predicted cognitive resilience in women.

## Methods

### Participants

The data were drawn from the Personality and Total Health Through Life (PATH) Study. Participants were randomly sampled from the electoral roll of the Australian Capital Territory and Queanbeyan in Australia and followed up over 12 years at 4-year intervals for a total of 4 waves. Participants engaged in cognitive assessment and self-report survey questions about their lifestyle, health and wellbeing. The details of this cohort are reported extensively elsewhere^32,33^. The study was conducted in accordance with the Declaration of Helsinki and was approved by the Human Ethics Committee of The Australian National University. All participants provided written informed consent before participating in the study. All methods were carried out in accordance with relevant guidelines and regulations.

This study examined participants who were aged 60+ at baseline (Mean age = 62.33, SD = 1.45, range: 60–65), had at least one *APOE ɛ4* allele and who underwent clinical assessment for MCI/dementia diagnosis at wave 4 (N = 341, 46% Women). Participants who had dementia at baseline (estimated using MMSE < 23) were excluded from analysis. Additionally, analysis of ethnicity showed that the sample was 97.69% Caucasian (with 2.31% being a mix of Aboriginal and Torres Strait Islander, Asian and other ethnicities) and thus the researchers decided not the include ethnicity as a predictor due to the lack of variability. This has been noted as a limitation in the discussion section.

### APOE status

Genotyping of the PATH sample has been described previously^34^. In summary, Qiagen Blood kits were used to extract genomic DNA from buccal swabs at baseline (wave 1). Overall, 90.1% of the 60s cohort provided buccal swabs. Genotypes of the two SNPs that define the *APOE* alleles (rs429358 and rs7412) were detected using TaqMan assays. The allele frequency distribution for each of the two SNPs was in accordance with the Hardy–Weinberg equilibrium denoting validity of the genotyping. *APOE* allele status was included in the analysis to examine the impact of the protective effect of *APOE ɛ2* (for participants who had *APOE ɛ2/ɛ4* combinations)^1^ and increased Alzheimer’s Disease risk associated with homozygous *APOE ɛ4* presentation (i.e. participants who have *APOE ɛ4/ɛ4*).

### Memory

Immediate recall was only assessed using one trial of the California Verbal Learning Test^35^ in wave 1 of PATH. While this increased to three trials in wave 4, only the first trial was used to maintain consistency for comparisons across the waves. The CVLT was conducted as part of a face-to-face cognitive test battery by a trained interviewer. Immediate recall score at wave 4 and the average slope across all 4 waves were used as variables in the in the resilience classification algorithm.

### Cognitive impairment status (MCI and dementia)

Cognitive impairment status was determined at wave 4 through clinical diagnosis. The process has been described previously^36^. Briefly, longitudinal assessment data were screened for cognitive impairment. Performance on the wave 4 cognitive battery was divided into neurocognitive domains. For each domain, the definition of MCI level of cognitive impairment was 1–1.5 SD below gender- and education-stratified norms (i.e. scores standardised relative to whole cohort sample at wave 4). For "dementia" level of impairment, 2.0 SD or more below gender- and education-stratified norms was used. The clinicians reviewed the cognitive profiles, along with informant reports, instrumental activities of daily living scales, self-reported medical conditions and MRI scans where necessary to determine diagnoses against DSM-5, DSM-IV and IWG criteria^37^. A consensus diagnosis including differentiation by dementia and MCI subtypes (using IWG criteria), was formed between a research neurologist and clinician specializing in psychiatry. In this paper, the variable indicating whether or not the participant had a diagnosis of cognitive impairment at wave 4 (specifically mild cognitive impairment or dementia based on DSM-IV or DSM-5 diagnostic criteria) was included in the resilience classification algorithm.

### Protective factors

Self-reported years of education (including total years of primary, secondary, post-secondary, and vocational education) was recorded at baseline along with mental activity, net positive social interactions, diet and physical activity. Mental activity was a composite measure of the RIASEC scales^38^ including the six domains of: Realistic, Investigative, Artistic, Social, Enterprising and Conventional activities. Net positive social interactions was number of positive social exchanges with family and friends minus negative exchanges collected via a self-report questionnaire^39^. A previous study showed that the MIND diet was protective against dementia in the PATH sample while the Mediterranean diet was not^40^. As such, we used the MIND diet as a protective factor in this study. The MIND diet was calculated based on food frequency questionnaire responses at baseline using the algorithm developed by Morris and colleagues^41^. This is a novel inclusion as none of the previous studies included diet in their analysis^12–15^. Frequency of physical activity was recorded as how often participants perform activities at each of the following three intensity levels: mild, moderate and vigorous activities. The scale was scored as follows: 1 = Never/hardly ever, 2 = About 1–3 times a month, 3 = Once or twice a week and 4 = 3 times a week or more (see^42^).

### Demographics

Age, gender, ethnicity (Caucasian/white, Aboriginal and Torres Strait Islander, Asian, other), self-reported relationship (partnered, not partnered) and employment status were recorded at baseline.

### Medical

Depression, anxiety, hypertension, diabetes, stroke, head injury, cholesterol medication and BMI were recorded at baseline. Depression and anxiety symptoms were measured with the Goldberg scale^43^. Participants were classified as hypertensive if their mean diastolic blood pressure was higher than 90 mmHg, or if their systolic blood pressure was higher than 140 mmHg, or if they were currently taking antihypertensive medication. Diabetes, stroke (including mini-stroke or transient ischemic attack), history of serious head-injury (with > 15 min loss of consciousness) and use of any medications for lowering cholesterol were recoded based on self-report. Body mass index (BMI) was calculated based on self-reported weight and measured height [weight(kg)/height^2^ (m^2^)].

### Lifestyle

Smoking (cigarettes per day) and alcohol consumption (drinks per week) were self-reported at baseline.

### Analysis

#### Group categorisation

Latent class analysis (LCA) was used to classify individuals into outcome groups based on memory performance and cognitive impairment status. Variables used in the LCA included memory at wave 4 (using residual wave 4 immediate recall scores after controlling for/regressing out age, gender and ethnicity), trajectories of memory decline (estimated using individual immediate recall slopes across the 4 waves) and cognitive impairment status at wave 4. Models ranging from one to three classes were examined using Mplus version 6.

In their study, Kaup et al.^12^ sought to answer the research question: “How do some *APOE ɛ4* carriers maintain cognitive health to the extent that their cognitive trajectories resemble those of the most cognitively healthy *APOE ɛ4* non-carriers?” As such, they characterised cognitive resilience based on the cognitive status of the entire cohort rather than just within *APOE ɛ4* carriers. They reasoned that comparing *APOE ɛ4* carriers with the entire cohort would yield a more representative classification of resilience than just basing their definition on *APOE ɛ4* carriers alone. We concur with this reasoning and thus performed our classification (LCA) on the whole 60+ PATH cohort rather than just within our sample of *APOE ɛ4* carriers. However, subsequent analyses post-classification focused only on *APOE ɛ4* carriers as this was our demographic of interest.

#### Comparisons between groups

Group comparisons were completed using SPSS v 25. ANOVA/*t* tests and Chi-square analysis was used to examine differences in baseline measures between the LCA groups among *APOE ɛ4* carriers. Logistic regression was used to examine the factors that predicted resilience. Predictors were separated into 3 models including: (1) protective factors, (2) genetic and demographics, and (3) medical and lifestyle factors. Model 1 (protective factors) included years of education, MIND diet score, physical activity, net positive social exchanges and cognitive activity. Model 2 (genetics and demographics) included *APOE ɛ4* allele status, age, partnered and employment status. Model 3 (medical and lifestyle) included hypertension, diabetes, heart problems, head injury, BMI, taking medication for cholesterol, anxiety, depression, number of cigarettes a day and number of drinks a week. Results were post-LCA were stratified by gender.

## Supplementary Information

Supplementary Information.

## Data Availability

PATH questionnaires are available through the PATH website: http://www.pathstudy.org.au/. PATH is not a publicly available dataset and it is not possible to gain access to the data without developing a collaboration with a PATH investigator. Researchers can access the data through an approval process by submitting a proposal to the PATH committee.
