# Supplementary material for: Gender specific factors contributing to cognitive resilience in APOE ɛ4 positive older adults in a population-based sample
Source: Sci Rep. 2023 May 17;13:8037. doi: 10.1038/s41598-023-34485-1 (PMC10192125; doi:10.1038/s41598-023-34485-1)
Supplement: Supplementary file 1 — Supplementary Information. [file 41598_2023_34485_MOESM1_ESM.docx]

**Appendix**

Contents

Appendix A - Latent Class Analysis Output 2

Appendix B - Descriptive statistics of LCA groups for non-APOE ɛ4 carriers and APOE ɛ4 carriers 4

Appendix C - Sensitivity Analysis including Individuals with Stroke 6

Appendix D - Vigorous Physical Activity by Occupation Category for Men 10

# **Appendix A - Latent Class Analysis Output**

To identify which model provided the best fit to the data, five fit indices were evaluated including the Bayesian Information Criterion (BIC; (1)), sample size adjusted BIC (SABIC; (2)), the Akaike Information Criterion (AIC; (3)), entropy (4) and the Lo-Mendell- Rubin Likelihood (5) ratio tests (LMR). Lower values of BIC, SABIC and AIC indicate better model fit. The SABIC and AIC also take into consideration the number of estimated parameters to decrease model complexity. Entropy values fall between 0 and 1. Entropy values near 0 indicate that the latent classes are not sufficiently well separated, or near chance separation (6). An entropy value closer to 1 indicates better categorisation. Finally, a significant result in the LMR tests indicate that model fit improves with the estimation of an additional class.

| Table A1. Latent Class Analysis Indices | | | | | |
| --- | --- | --- | --- | --- | --- |
| Number of classes | AIC | BIC | SABIC | Entropy | LMR |
| 1 | 14308.809 | 14337.279 | 14321.393 |  |  |
| 2 | 13966.653 | 14017.898 | 13989.304 | 0.535 | <.001 |
| 3 | 13792.051 | 13866.072 | 13824.769 | 0.611 | 0.045 |

**
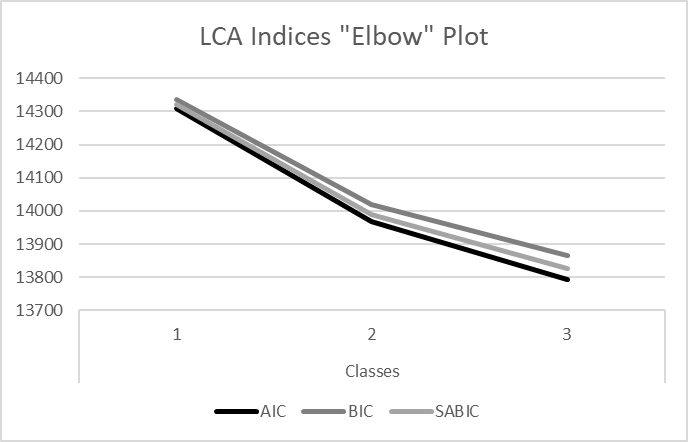
**

**References**

1. Schwarz G. Estimating the dimension of a model. The annals of statistics. 1978:461-4.

2. Sclove SL. Application of model-selection criteria to some problems in multivariate analysis. Psychometrika. 1987;52(3):333-43.

3. Akaike H. Factor analysis and AIC. Selected papers of hirotugu akaike: Springer; 1987. p. 371-86.

4. Ramaswamy V, DeSarbo WS, Reibstein DJ, Robinson WT. An empirical pooling approach for estimating marketing mix elasticities with PIMS data. Marketing science. 1993;12(1):103-24.

5. Lo Y, Mendell NR, Rubin DB. Testing the number of components in a normal mixture. Biometrika. 2001;88(3):767-78.

6. Masyn KE. 25 latent class analysis and finite mixture modeling. Oxford University Press; 2013. p. 551.

# **Appendix B - Descriptive statistics of LCA groups for non-APOE ɛ4 carriers and APOE ɛ4 carriers**

| Table B1. Descriptive statistics of LCA groups for non-*APOE ɛ4* carriers and *APOE ɛ4* carriers | | | | |
| --- | --- | --- | --- | --- |
|  | Non-*APOE ɛ4* carrier (N = 863) | *APOE ɛ4* carrier (N = 341) | T-value (Cohen’s d)/ Chi-squared (Cramer’s V) | P value |
| Mean Age (wave 1) | 62.59 (1.51) | 62.34 (1.46) | 1.441  (0.087) | .011 |
| Immediate recall (wave 1) | 7.50 (2.12) | 7.31 (2.31) | -2.214  (-0.145) | .179 |
| Immediate recall (wave 4) | 5.56 (1.89) | 5.30 (1.96) | -33.773  (-2.472) | .066 |
| Dementia/MCI diagnoses (wave 4) | 104 (15.4%) | 61 (22.7%) | 7.047  (0.086) | .008 |
| Years of education (wave 1) | 14.09 (2.60) | 14.29 (2.50) | -4.661  (-0.288) | .215 |
| Women | 442 (51.2%) | 158 (46.3%) | 2.331  (0.044) | .127 |
| Partnered (wave 1) | 692 (80.2%) | 289 (84.8%) | 3.376  (0.053) | .066 |
| Employed (wave 1) | 345 (40.0%) | 143 (41.9%) | 0.389  (0.018) | .533 |
| Hypertension (wave 1) | 543 (62.9%) | 210 (61.6%) | 0.186  (0.012) | .666 |
| Cholesterol Medication (wave 1) | 180 (20.9%) | 86 (25.2%) | 2.703  (0.047) | .100 |
| Diabetes (wave 1) | 796 (92.2%) | 312 (91.5%) | 0.183  (0.012) | .669 |
| Heart Problems (wave 1) | 124 (14.4%) | 45 (13.2%) | 0.278  (0.015) | .598 |
| Head Injury (wave 1) | 42 (4.9%) | 14 (4.1%) | 0.319  (0.016) | .572 |
| BMI (wave 1) | 26.79 (5.26) | 26.35 (4.68) | 1.395  (0.087) |  |
| MIND diet (wave 1) | 6.33 (1.40) | 6.49 (1.42) | -2.075  (-0.129) | .082 |
| Frequency of Physical Activity (wave 1) |  |  |  |  |
| Mild | 3.61 (0.74) | 3.65 (0.72) | -0.269  (-0.017) | .448 |
| Moderate | 2.94 (0.97) | 2.90 (1.02) | -1.308  (-0.081) | .549 |
| Vigorous | 1.71 (1.01) | 1.69 (0.97) | -0.844  (-0.052) | .831 |
| Anxiety score (wave 1) | 2.01 (2.17) | 2.05 (2.21) | 1.659  (0.103) | .742 |
| Depression score (wave 1) | 1.46 (1.73) | 1.44 (1.68) | 2.455  (0.143) | .847 |
| Number of cigarettes per day (wave 1) | 0.94 (4.11) | 1.17 (5.14) | -0.978  (-0.067) | .400 |
| Number of drinks per week (wave 1) | 7.08 (8.69) | 6.40 (6.75) | -1.125  (-0.070) | .150 |
| Net positive social interactions (wave 1) | 12.10 (7.26) | 11.85 (7.82) | -0.264  (-0.016) | .602 |
| Mental activity (wave 1) | 22.60 (7.42) | 22.91 (7.28) | -3.679  (-0.219) | .510 |

# **Appendix C - Sensitivity Analysis including Individuals with Stroke**

| Table C1. Bivariate Comparisons and Logistic Regression Results for Men Including Those with Stroke (N = 183) | | | | | | | |
| --- | --- | --- | --- | --- | --- | --- | --- |
| T-Test/Chi-Squared | | | | | Odds ratio† (95% CI) | | |
| Variables | Resilient (N = 48) | Non-resilient (N = 135) | T-value (Cohen’s d)/ Chi-squared (Cramer’s V) | p-value | Model 1 (Protective Factors only) | Model 2 (Including demographic and genetic risk factors) | Model 3 (Including medical and lifestyle risk factors) |
| Years of education (wave 1) | **15.49 (2.43)** | **14.29 (2.56)** | **-2.831**  **(-0.476)** | **.005** | **1.215 (1.03; 1.432)*** | **1.221 (1.031; 1.446)*** | **1.268 (1.043; 1.542)*** |
| MIND diet score | 6.64 (1.45) | 6.17 (1.47) | -1.898  (-0.319) | .059 | 1.121 (0.857; 1.467) | 1.172 (0.882; 1.556) | 1.209 (0.882; 1.658) |
| Frequency of Physical Activity (wave 1) |  |  |  |  |  |  |  |
| Mild | 3.73 (0.64) | 3.58 (0.76) | -1.334  (-0.207) | .185 | 1.508 (0.816; 2.785) | 1.799 (0.946; 3.42) | **2.141 (1.08; 4.245)*** |
| Moderate | 3.1 (0.83) | 3.1 (0.88) | -.003  (-0.001) | .997 | 0.978 (0.615; 1.556) | 0.882 (0.548; 1.42) | 0.907 (0.539; 1.524) |
| Vigorous | **1.52 (0.8)** | **1.92 (1.04)** | **2.721**  **(0.403)** | **.008** | **0.570 (0.369; 0.88)*** | **0.544 (0.349; 0.847)*** | **0.503 (0.309; 0.817)*** |
| Positive Social Interactions (wave 1) | 12.83 (6.75) | 13.03 (7.34) | 0.162  (0.027) | .871 | 1.006 (0.957; 1.058) | 1.016 (0.962; 1.074) | 1.018 (0.951; 1.09) |
| Mental activity (wave 1) | 25.52 (7.02) | 24.39 (7.34) | -0.931  (-0.156) | .353 | 1.002 (0.951; 1.057) | 0.994 (0.938; 1.053) | 1.006 (0.943; 1.074) |
| Age (wave 1) | 62.52 (1.49) | 62.32 (1.54) | -0.789  (-0.133) | .431 | - | 1.104 (0.871; 1.398) | 1.088 (0.842; 1.407) |
| Partnered (wave 1) | 45 (93.8%) | 126 (93.3%) | 0.010  (0.007) | .920 | - | 0.510 (0.095; 2.739) | 0.444 (0.076; 2.588) |
| Employed (wave 1) | 25 (52.1%) | 58 (43%) | 1.188  (0.081) | .276 | - | 1.854 (0.855; 4.023) | 2.158 (0.93; 5.01) |
| *APOE Status* |  |  |  |  | - |  |  |
| *APOE ɛ2/ɛ4* | 4 (8.3%) | 12 (9.6%) | 3.526  (0.139) |  | - | 0.539 (0.15; 1.931) | 0.569 (0.142; 2.287) |
| *APOE ɛ4/ɛ4* | 0 | 9 (6.7%) |  | .172 | - | 0 | 0 |
| Hypertension (wave 1) | 35 (72.9%) | 88 (65.2%) | 0.960  (0.072) | .327 | - | - | 2.108 (0.834; 5.325) |
| Cholesterol Medication (wave 1) | 16 (33.3%) | 34 (25.2%) | 1.184  (0.080) | .277 | - | - | 1.033 (0.401; 2.66) |
| Diabetes (wave 1) | 1 (2.1%) | 12 (8.9%) | 2.485  (0.117) | .115 | - | - | 0.192 (0.021; 1.783) |
| Heart Problems (wave 1) | 9 (18.8%) | 21 (15.6%) | 0.264  (0.038) | .608 | - | - | 1.550 (0.5; 4.804) |
| Head Injury (wave 1) | 2 (4.2%) | 10 (7.4%) | 0.607  (0.058) | .436 | - | - | 0.400 (0.06; 2.664) |
| BMI (wave 1) | 26.27 (3.23) | 26.47 (3.95) | 0.307  (0.052) | .759 | - | - | 0.916 (0.816; 1.028) |
| Anxiety score (wave 1) | 2.04 (2.45) | 1.81 (1.96) | -0.579  (-0.108) | .564 | - | - | 1.086 (0.834; 1.416) |
| Depression score (wave 1) | 1.33 (1.68) | 1.37 (1.64) | 0.133  (0.022) | .894 | - | - | 1.059 (0.76; 1.476) |
| Number of cigarettes per day (wave 1) | 1.52 (5.61) | 1.11 (4.87) | -0.480  (-0.081) | .631 | - | - | 1.058 (0.972; 1.15) |
| Number of drinks per week (wave 1) | 7.94 (6.88) | 8.15 (7.72) | 0.165  (0.028) | .869 | - | - | 0.963 (0.908; 1.022) |

*p<.05. †Non-resilient group used as reference group. Model 2 includes *APOE ɛ4* allele status, age, partnered and employment status. Model 3 includes hypertension, diabetes, heart problems, head injury, BMI, cholesterol medication, anxiety, depression, smoking and alcohol consumption.

| Table C2. Bivariate Comparisons and Logistic Regression Results for Women Including Those with Stroke (N = 158) | | | | | | | |
| --- | --- | --- | --- | --- | --- | --- | --- |
| T-Test/Chi-squared | | | | | Odds ratio† (95% CI) | | |
| Variables | Resilient (N = 53) | Non-resilient (N = 105) | T-value (Cohen’s d)/ Chi-squared (Cramer’s V) | p-value | Model 1 (Protective Factors only) | Model 2 (Including demographic and genetic risk factors) | Model 3 (Including medical and lifestyle risk factors) |
| Years of education (wave 1) | **14.51 (2.22)** | **13.63 (2.4)** | **-2.222**  **(-0.374)** | **.028** | 1.132 (0.965; 1.328) | 1.088 (0.92; 1.286) | 1.086 (0.91; 1.297) |
| MIND diet score | 6.86 (1.45) | 6.64 (1.22) | -1.003  (0.169) | .318 | 1.079 (0.813; 1.432) | 1.111 (0.827; 1.491) | 1.151 (0.837; 1.582) |
| Frequency of Physical Activity (wave 1) |  |  |  |  |  |  |  |
| Mild | 3.72 (0.66) | 3.66 (0.73) | -0.501  (-0.084) | .617 | 0.999 (0.576; 1.733) | 1.029 (0.579; 1.828) | 0.782 (0.42; 1.456) |
| Moderate | 2.66 (1.14) | 2.66 (1.12) | -0.017  (-0.003) | .986 | 0.871 (0.611; 1.24) | 0.884 (0.618; 1.265) | 0.863 (0.586; 1.269) |
| Vigorous | 1.64 (0.98) | 1.5 (0.88) | -0.888  (-0.150) | .376 | 1.270 (0.842; 1.916) | 1.280 (0.839; 1.953) | 1.347 (0.841; 2.156) |
| Positive Social Interactions (wave 1) | 11.4 (9.21) | 10.11 (7.89) | -0.911  (-0.153) | .364 | 1.018 (0.974; 1.063) | 0.998 (0.949; 1.048) | 0.995 (0.942; 1.052) |
| Mental activity (wave 1) | **22.38 (5.6)** | **20.09 (7.2)** | **-2.199**  **(-0.341)** | **.030** | 1.041 (0.986; 1.099) | 1.041 (0.984; 1.101) | 1.057 (0.993; 1.125) |
| Age (wave 1) | 62.23 (1.35) | 62.34 (1.41) | 0.498  (0.084) | .620 | - | 0.908 (0.701; 1.177) | 0.934 (0.705; 1.237) |
| Partnered (wave 1) | 44 (83%) | 74 (70.5%) | 2.931  (0.136) | .087 | - | 2.062 (0.784; 5.422) | 2.515 (0.842; 7.511) |
| Employed (wave 1) | 23 (43.4%) | 37 (35.2%) | 0.995  (0.079) | .318 | - | 1.190 (0.558; 2.538) | 1.024 (0.454; 2.306) |
| *APOE* Status |  |  |  |  | - |  |  |
| *APOE ɛ2/ɛ4* | 7 (13.2%) | 10 (9.5%) | 2.766  (0.132) |  | - | 1.440 (0.489; 4.238) | 1.639 (0.531; 5.061) |
| *APOE ɛ4/ɛ4* | 5 (9.4%) | 4 (3.8%) |  | .251 | - | 2.449 (0.545; 11.011) | 1.818 (0.387; 8.536) |
| Hypertension (wave 1) | 28 (52.8%) | 59 (56.2%) | 0.161  (0.032) | .688 | - | - | 1.100 (0.507; 2.389) |
| Cholesterol Medication (wave 1) | 10 (18.9%) | 26 (24.8%) | 0.695  (0.066) | .404 | - | - | 0.766 (0.285; 2.057) |
| Diabetes (wave 1) | 5 (9.4%) | 11 (10.5%) | 0.042  (0.016) | .838 | - | - | 0.617 (0.157; 2.429) |
| Heart Problems (wave 1) | 3 (5.7%) | 12 (11.4%) | 1.364  (0.093) | .243 | - | - | 0.306 (0.071; 1.318) |
| Head Injury (wave 1) | 0 | 2 (1.9%) | 1.022  (0.080) | .312 | - | - | 0 |
| BMI (wave 1) | 26.74 (6.95) | 26.05 (4.72) | -0.738  (-0.124) | .462 | - | - | 1.050 (0.97; 1.137) |
| Anxiety score (wave 1) | 1.81 (2.14) | 2.49 (2.37) | 1.741  (-0.124) | .084 | - | - | 0.883 (0.697; 1.12) |
| Depression score (wave 1) | **1.17 (1.42)** | **1.71 (1.83)** | **2.054**  **(0.319)** | **.042** | - | - | 0.959 (0.67; 1.372) |
| Number of cigarettes per day (wave 1) | 1.42 (7.36) | 0.98 (3.76) | -0.491  (-0.083) | .824 | - | - | 1.055 (0.974; 1.144) |
| Number of drinks per week (wave 1) | 5.44 (5.47) | 3.93 (4.93) | -1.754  (-0.295) | .081 | - | - | 1.042 (0.967; 1.122) |

*p<.05. †Non-resilient group used as reference group. Model 2 includes *APOE ɛ4* allele status, age, partnered and employment status. Model 3 includes hypertension, diabetes, heart problems, head injury, BMI, cholesterol medication, anxiety, depression, smoking and alcohol consumption.

# **Appendix D - Vigorous Physical Activity by Occupation Category for Men**

Data were obtained from PATH wave 1 60s Questionnaire. Occupations are coded based on ANZSCO major group codes.

| Table D1. Vigorous Physical Activity by Occupation Category for Men | | | |
| --- | --- | --- | --- |
| **Frequency of Vigorous Physical Activity** | **Occupation Category**  *(Occupation Missing N = 1)* | | **Total** |
|  | **Non-laborious roles** | **Laborious roles** |  |
| **Never / hardly ever** | 61 (49%) | 19 (54%) | 80 |
| **About 1-3 times a month** | 37 (30%) | 6 (17%) | 43 |
| **Once or twice a week** | 16 (13%) | 6 (17%) | 22 |
| **3 times a week or more** | 11 (9%) | 4 (11%) | 15 |
| **Total** | 125 | 35 | 160 |

*Non-laborious roles = Managers, Professionals, Community and Personal Service Workers, Clerical and Administrative Workers, Sales Workers*

*Laborious roles = Technicians and Trades Workers, Machinery Operators and Drivers, Labourers*
